# Adrenalectomy as a Treatment Option for Primary Aldosteronism in the Era of Robotic-Assisted Surgeries—Is It Time to Use It More Often?

**DOI:** 10.3390/jcm15010173

**Published:** 2025-12-25

**Authors:** Orit Raz, Naomi Nakash Niddam, Fahed Atamna, Alla Simonovsky, Sergey Litvin, Mia Leonov Polak, Adi Leiba, Dor Golomb

**Affiliations:** 1 Department of Urology, Samson Assuta Ashdod University Hospital, 7 Harefua St., Ashdod 7747629, Israel; oritra@assuta.co.il (O.R.); faheda@assuta.co.il (F.A.); 2 Department of Nephrology, Samson Assuta Ashdod University Hospital, Ashdod 7747629, Israel; naomin@assuta.co.il (N.N.N.); adilei@assuta.co.il (A.L.); 3 Department of Diagnostic Radiology, Samson Assuta Ashdod University Hospital, Ashdod 7747629, Israel; allas@assuta.co.il; 4 Department of Interventional Radiology, Samson Assuta Ashdod University Hospital, Ashdod 7747629, Israel; sergeyl@assuta.co.il; 5 Department of Pathology, Samson Assuta Ashdod University Hospital, Ashdod 7747629, Israel; mial@assuta.co.il

**Keywords:** adrenal, primary aldosteronism, robotic adrenalectomy

## Abstract

**Objectives:** To evaluate clinical and biochemical outcomes of robotic-assisted laparoscopic adrenalectomy in patients with primary aldosteronism (PA) due to small aldosterone-producing adenomas, with emphasis on blood pressure (BP) control, antihypertensive medication burden, hormonal normalization, and safety. **Methods:** We prospectively enrolled PA patients (aldosterone >10 ng/dL, renin <2 μU/mL) undergoing robotic adrenalectomy by a single surgeon. Exclusions included suspected pheochromocytoma, other adrenal pathologies, or malignancy. Outcomes were classified per PASO criteria at 6 months: *complete success* (BP <140/90 mmHg without medications + normalized aldosterone (<10 ng/dL) and renin (>2 μU/mL)), *partial success* (improvement in BP control with reduced medication and/or partial biochemical improvement), and *failure* (persistent hypertension and abnormal hormone levels). **Results:** From 2019 to present, 18 patients (median age 53 years; 13 male) with a median adenoma size of 15 mm (IQR 10–19.8) underwent robotic adrenalectomy (12 left, 6 right). Three (16.7%) with bilateral imaging findings had adrenal vein sampling to confirm unilateral disease. At 6 months, complete clinical success was achieved in 10 (55.5%) patients, partial success in 7 (38.9%), and failure in 1 (5.6%). Biochemically, 12 achieved complete normalization, 3 achieved partial improvement, and 3 did not complete testing. Median operative time was 110 min (IQR 100–120); median hospital stay was 3 days (IQR 3–4). No intra- or postoperative complications, transfusions, infections, or readmissions occurred. **Conclusions:** Robotic adrenalectomy for small aldosterone-producing adenomas in PA is safe, with high rates of BP normalization and hormonal remission and significantly reduced antihypertensive medication burden.

## 1. Introduction

PA is the most common cause of secondary hypertension, resulting from autonomous overproduction of aldosterone by one or both adrenal glands. It affects an estimated 5% of all patients with hypertension, increasing to 10% in referred populations and up to 20% among those with treatment-resistant hypertension [1]. Beyond its impact on BP, PA is associated with significant morbidity due to its profibrotic and proinflammatory effects, leading to cardiovascular, cerebrovascular, and renal complications and contributing to excess mortality if left untreated [2]. PA is typically classified into unilateral and bilateral forms, which require distinct therapeutic approaches. Unilateral disease, frequently due to an aldosterone-producing adenoma with benign imaging features, is potentially curable through adrenalectomy, whereas bilateral adrenal hyperplasia is generally managed with mineralocorticoid receptor antagonists [3].

**Figure 1:**
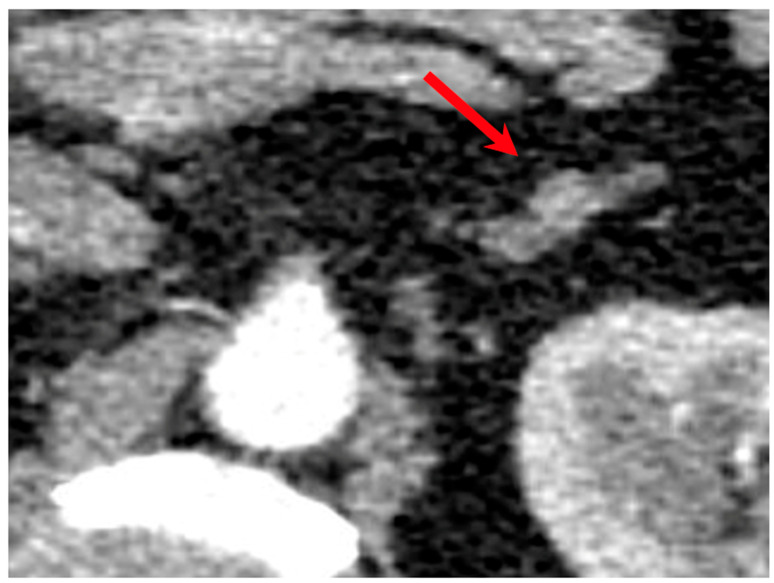
CT scan of an 8 mm left adrenal adenoma. Red arrow indicates the adenoma.

**Figure 2:**
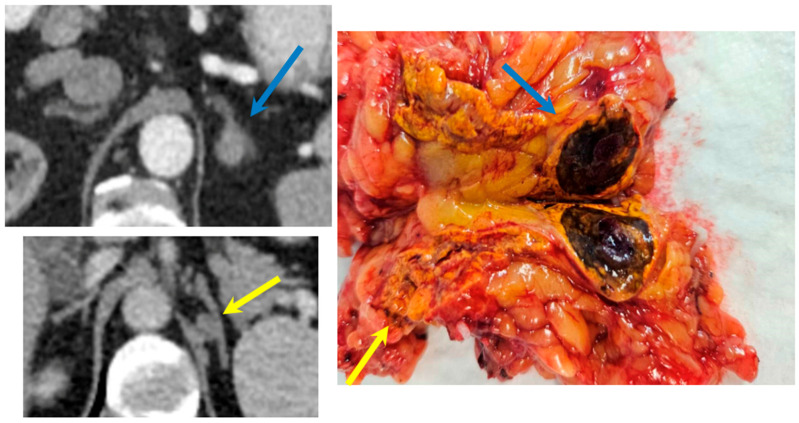
Surgical specimen showing a 15 mm adenoma located in the posterior horn of the left adrenal gland (blue arrow) and an 11 mm adenoma in the superior posterior horn of the left adrenal gland (yellow arrow) and CT scan and surgical specimen of the right adrenal gland displaying a 25 mm adenoma in the anterior horn (yellow arrow) and an additional 8 mm adenoma in the posterior horn (blue arrow).

Unfortunately, the diagnosis of PA is often delayed or missed [4]. Patients typically undergo prolonged treatment in primary care with escalating antihypertensive regimens—including multiple drug classes—before eventual referral to a nephrologist. Even then, many specialists may lack an adrenal-focused diagnostic approach. Imaging often reveals small adrenal nodules (Figure 1), two in the same adrenal gland (Figure 2), or bilateral adenomas with benign characteristics that remain stable in size over time, further contributing to under-recognition. Although adrenal vein sampling (AVS) remains the gold standard for subtype classification and lateralization of aldosterone secretion [5], its technical complexity and limited availability often hinder its routine implementation. In many cases, AVS is not feasible due to anatomical challenges, particularly when selective cannulation of the adrenal veins via angiography fails [6].

While medical therapy with mineralocorticoid receptor antagonists is an effective management strategy, adrenalectomy represents the only potentially curative option for eligible patients with unilateral PA, many of whom are relatively young yet severely affected [7]. Biochemical cure is defined as normalization of aldosterone, renin, and potassium levels. However, clinical cure, defined as complete normalization of BP without the need for hypertensive medications, remains more variable. Notably, patients may continue to experience essential hypertension despite biochemical remission, highlighting the need to evaluate both clinical and biochemical outcomes distinctly, as persistent hypertension may be due to other coexisting causes of hypertension.

Since its introduction in 1999, robot-assisted laparoscopic adrenalectomy has gained popularity due to its technical advantages over conventional laparoscopy [8]. These include enhanced ergonomics, superior three-dimensional visualization, tremor filtering, and increased degrees of freedom provided by the robotic instruments. These features are particularly beneficial in adrenal surgery, where the delicate gland is located deep and high in the retroperitoneum, surrounded by major vessels and visceral organs. The robotic approach facilitates precise dissection in this confined and high-risk area, thereby reducing intraoperative complications and potentially improving surgical outcomes. An expert urology robotic surgeon is able to handle this delicate surgery with three robotic arms, while controlling the camera and dissecting the adrenal from the renal vessels, aorta on the left, and inferior vena cava (IVC) in right-sided adrenalectomy.

This study aims to evaluate the impact of robot-assisted laparoscopic transperitoneal adrenalectomy in patients with aldosterone-producing adenomas. Specifically, we assess its association with BP reduction, decreased reliance on antihypertensive medications, and normalization of aldosterone-to-renin ratios. Additionally, we examine the rate of perioperative complications, such as bleeding, infection, and readmission. Given the severity of disease burden in this population, the observed clinical and biochemical improvements—achieved with the minimally invasive robotic approach—may offer substantial benefits in terms of morbidity reduction, quality of life, and long-term cardiovascular protection.

## 2. Methods

After obtaining institutional review board approval (0002-25-AAA) and adhering to the 2013 revision of the World Medical Association Declaration of Helsinki, we performed a prospective cohort study of patients with aldosterone-producing adenoma undergoing unilateral robotic-assisted laparoscopic adrenalectomy. The diagnostic workup was performed in accordance with the Endocrine Society Clinical Practice Guidelines. Screening was conducted using the aldosterone-to-renin ratio (ARR). Patients with an elevated ARR underwent confirmatory testing, typically utilizing the Saline Infusion Test. In adherence to guideline recommendations, confirmatory testing was bypassed only in patients presenting with spontaneous hypokalemia, undetectable renin levels, and markedly elevated aldosterone concentrations. Renin levels were assessed using a Direct Renin Concentration (DRC) assay. Inclusion criteria were adult patients (≥18 years) with confirmed PA. All were surgical candidates fit for anesthesia and consented to data collection. Exclusion criteria included suspected pheochromocytoma, other adrenal pathologies (e.g., Cushing’s and carcinoma), malignancy, surgical contraindications, or non-adenoma unilateral causes. Data were collected prospectively, encompassing preoperative laboratory and imaging results, surgical details, and postoperative outcomes. All operations employed a standardized robotic-assisted laparoscopic transperitoneal approach. Antihypertensive medication usage was measured via defined daily doses (DDD). At 6 months post-adrenalectomy, clinical and biochemical outcomes were evaluated using the 2017 Primary Aldosteronism Surgical Outcome (PASO) consensus criteria, distinguishing between the two categories. Clinical outcomes were classified as complete success (blood pressure <140/90 mmHg without antihypertensive medications), partial success (reduced blood pressure or decreased medication count while attaining normal blood pressure), or absent success (unchanged or worsened blood pressure or medication needs). Biochemical outcomes were classified as complete success (normalized aldosterone-to-renin ratio with aldosterone <10 ng/dL and renin >2 μU/mL), partial success (improved but incompletely normalized parameters), or absent success (unchanged or worsened parameters).

### 2.1. Statistical Analysis

Descriptive statistics were used to summarize patient demographics, clinical characteristics, and outcomes. Continuous variables such as age, BMI, adenoma size, and number of postoperative antihypertensive medications are reported as medians with ranges. Categorical variables are summarized as counts and percentages. Comparisons between male and female patients were conducted using the Mann–Whitney U test for continuous variables and Fisher’s exact test for categorical variables. A *p*-value of <0.05 was considered statistically significant.

### 2.2. Surgical Technique

Our study employed robotic-assisted laparoscopic transperitoneal adrenalectomy using the Hugo™ Robotic-Assisted Surgery (RAS) system (Medtronic, Minneapolis, MN, USA), with patients positioned in the lateral decubitus position (operative side up) and flexed at the waist to optimize exposure, while port placement mirrored that for upper pole partial nephrectomy (Supplementary Figure S1) and included an additional 5 mm subxiphoid assistant port for liver retraction with a fan retractor in right-sided cases. The procedure began with medial mobilization of the colon (descending on the left, ascending/hepatic flexure on the right) to access the retroperitoneum, division of splenorenal or hepatophrenic ligaments as needed, incision of Gerota’s fascia along the upper lateral kidney to expose the upper pole, and cranial extension to separate the adrenal vein from the spleen or liver, using the renal artery pulse as a landmark for the cranial hilum. Key steps involved early isolation and clipping (with Hem-o-lok or metal clips) of the main adrenal vein (to the renal vein on the left or directly to the IVC on the right) to minimize gland manipulation, circumferential periadrenal fat dissection with bipolar energy for hemostasis from cranial to medial borders while avoiding major vessels, lateral lifting of the gland for retrograde dissection of medial attachments with posterior retroperitoneal exploration for accessory vessels, and specimen retrieval in a bag followed by hemostasis confirmation and routine retroperitoneal drain placement. Technical challenges, particularly on the right side due to the short adrenal vein, direct IVC drainage, and liver retraction needs—which heighten risks of vascular injury or bleeding—are mitigated by the robotic platform’s 3D visualization, wristed instruments, and tremor filtration, proving especially advantageous in obese patients with abundant fat or for precise excision of small adenomas without fragmentation.

## 3. Results

From September 2019 to present, 18 patients with confirmed PA underwent robotic-assisted laparoscopic adrenalectomy at our center. Demographic and clinical details are summarized in Table 1. The cohort comprised 13 (72.2%) males and 5 (27.8%) females, with a median age of 53 years (IQR 44–65). All patients presented with treatment-resistant hypertension and elevated aldosterone-to-renin ratios. The median adenoma size was 15 mm (IQR 10–19.8). Three (16.7%) patients with bilateral adrenal lesions underwent adrenal vein sampling before surgery to confirm unilateral disease. Left adrenalectomy was performed in 12 (66.6%) cases, and right adrenalectomy in 6 (33.3%) cases. The median operative time was 110 min (IQR 100–120), and the median hospital stay was 3 days (IQR 3–4). No intraoperative or postoperative complications, including bleeding, infections, or readmissions, were observed. All resected adrenal specimens underwent histopathological examination, which confirmed the presence of adrenocortical adenomas consistent with aldosterone overproduction in all 18 patients. There were no cases of unexpected malignancy or discordance between the preoperative imaging, clinical, and pathological findings. For clinical outcomes, complete success was achieved in 10 patients (55.5%), who ceased all antihypertensive medications and achieved normal BP. Partial success was observed in seven patients (38.9%), including one patient requiring one antihypertensive medication, four patients requiring two antihypertensive medications, and two patients requiring three antihypertensive medications; all had normal BP and reduced medication burden with BP control compared to preoperative status (median three antihypertensive medications, IQR 2–4). One patient had absent clinical success, failing to improve biochemical profile and with high BP. For biochemical outcomes, complete success was achieved in 12 patients, with normalized aldosterone (<10 ng/dL) and renin (>2 μU/mL) levels. Partial success was observed in three patients, who demonstrated partial biochemical improvement (including two partial clinical responses and one clinical failure). Three patients did not complete an updated biochemical profile, 6 months postoperatively. These outcomes are summarized in Table 2.

## 4. Discussion

This study examines the relationship between robotic-assisted laparoscopic transperitoneal adrenalectomy and postoperative changes in BP and antihypertensive medication use in a consecutive cohort of patients treated for PA at our institution. The robotic platform offers significant advantages over conventional laparoscopy, including superior ergonomics, high-definition three-dimensional visualization, tremor suppression, and greater instrument dexterity utilizing the open console Hugo^TM^ Ras Robotic-assisted surgery system by Medtronic. These features make the robotic approach particularly well-suited for adrenalectomy, given the adrenal gland’s delicate structure and its anatomy location within a confined space bordered by major vessels and visceral organs, where inadvertent injury could have severe clinical consequences.

Beyond the general technical advantages of enhanced dexterity and superior visualization, we propose that robotic-assisted adrenalectomy is particularly advantageous in specific patient and anatomical scenarios where precision is paramount and the tumor is not excessively large. The 3D visualization and wristed instruments are highly beneficial for right-sided adrenalectomies, enabling meticulous and safe dissection around the delicate inferior vena cava (IVC). Furthermore, the ergonomic benefits are pronounced in patients with higher Body Mass Index (BMI), where the robotic arms can navigate the substantial retroperitoneal fat pad with greater mechanical stability than conventional laparoscopic instruments. Finally, the magnified view is ideal for targeting small adenomas (such as the median 15 mm size in our cohort), ensuring complete excision while preserving surrounding tissue integrity. These patient and tumor characteristics represent specific indications where the added technical capability of the robotic platform offers clear clinical value over conventional laparoscopy. Our results emphasize the high yield of robotic-assisted adrenalectomy in unilateral PA, achieving high success rates in the majority of patients. Though 8 patients still required some antihypertensive medications postoperatively, they all experienced a significant and clinically relevant decline in both BP and medication burden, underscoring the therapeutic value of adrenalectomy in this population. Persistent BP post-adrenalectomy may reflect other factors affecting BP, as well as a delay in biochemical improvement. In some cases, it may require a longer period after removing the adenoma for biochemical resolution. Reduction in BP and decreased reliance on antihypertensive medications, alongside biochemical resolution, represent clinically meaningful outcomes of robotic-assisted laparoscopic transperitoneal adrenalectomy in patients with primary aldosteronism. While our cohort demonstrated excellent operative and clinical outcomes, we acknowledge the reviewer’s comment regarding the analysis of factors influencing these results. Due to the relatively small sample size, our study was not adequately powered to perform robust multivariate analysis to identify independent predictors of operative metrics (e.g., operative time, blood loss) or clinical success. However, in a preliminary review of our data, we did not observe a statistically significant difference in operative time between left-sided versus right-sided adrenalectomies, nor a strong correlation between smaller adenoma size and operative duration.

Hypertension that is unresponsive to standard pharmacological treatment may be a manifestation of underlying PA, a frequently underdiagnosed cause of secondary hypertension. PA is characterized by autonomous aldosterone secretion, leading to sodium retention, potassium excretion, and plasma volume expansion, which collectively contribute to persistently elevated BP despite multidrug regimens. Patients with PA often require multiple classes of antihypertensive medications yet continue to exhibit inadequate BP control, highlighting the need for etiologic evaluation. If left untreated, PA is associated with significantly greater risks of cardiovascular events, including myocardial infarction, stroke, atrial fibrillation, and chronic kidney disease, compared to patients with essential hypertension matched for BP levels [1]. These complications stem not only from sustained hypertension but also from the direct deleterious effects of aldosterone on vascular and myocardial tissue. Therefore, timely diagnosis and targeted treatment—particularly surgical resection in unilateral disease—are critical to mitigate long-term morbidity and mortality associated with this condition.

The diagnosis of primary aldosteronism is frequently delayed, in part due to its clinical overlap with essential hypertension and the prevailing tendency to escalate pharmacologic treatment rather than pursue etiologic investigation [4]. Many patients with PA undergo prolonged periods of suboptimal management, often prescribed multiple classes of antihypertensive medications by primary care physicians without further diagnostic workup. Referral to a specialist is typically prompted only after sustained treatment resistance, and even then, the evaluation may not include adrenal-focused diagnostics. This delay is compounded by the subtle imaging findings commonly associated with PA—small adrenal nodules with benign characteristics that are easily overlooked or deemed incidental. Consequently, adrenal imaging is often deferred until late in the clinical course, and the discovery of an aldosterone-producing adenoma may come only after years of poorly controlled hypertension and accumulating end-organ damage. These diagnostic delays not only perpetuate unnecessary morbidity but also defer access to potentially curative surgical intervention, highlighting the need for increased awareness and earlier screening for PA in patients with treatment-resistant hypertension. Importantly, many patients affected by PA are relatively young yet suffer from severe hypertension and its complications at an early age [1]. These patients often present with significant cardiovascular and metabolic comorbidities despite their age, underscoring the aggressive nature of aldosterone-mediated disease. For patients with unilateral PA due to an aldosterone-producing adenoma, surgical adrenalectomy remains the only potentially curative treatment. Unlike medical therapy, which manages symptoms but does not eliminate the source of hormone excess, adrenalectomy offers the prospect of complete biochemical and clinical remission. As such, timely identification and appropriate surgical intervention are critical to preventing long-term end-organ damage and improving quality of life in this vulnerable patient population.

The robotic surgical approach offers several distinct advantages over both open and conventional laparoscopic adrenalectomy, making it particularly well-suited for the treatment of PA. Compared to open surgery, the robotic technique is associated with reduced postoperative pain, shorter hospital stays, faster recovery, and fewer wound-related complications [9]. Relative to conventional laparoscopy, robotic-assisted adrenalectomy provides superior three-dimensional visualization, tremor filtration, and enhanced instrument dexterity through articulated EndoWrist tools, allowing for precise dissection of the adrenal gland in its confined retroperitoneal space adjacent to major vascular structures. These technical benefits are especially important in PA, where the adrenal lesions are often small and surrounded by critical anatomy. Our institution has achieved a high rate of both clinical and biochemical success using the robotic approach, with minimal perioperative complications. This underscores the reproducibility, safety, and effectiveness of robotic adrenalectomy for PA.

The high success rate of robotic-assisted laparoscopic adrenalectomy in our cohort underscores its potential as a transformative approach for managing unilateral PA, particularly in the context of evolving surgical technologies. The precision and minimally invasive nature of the robotic platform not only enhance surgical outcomes but also align with broader trends in personalized medicine, where tailored interventions can significantly improve patient quality of life. Furthermore, the absence of major perioperative complications in our study highlights the safety profile of this approach, which is critical for encouraging its adoption in centers with adequate robotic expertise. As robotic systems continue to advance, with improvements in haptic feedback and artificial intelligence-driven surgical planning, the efficacy and accessibility of robotic adrenalectomy may further increase. However, to fully establish robotic adrenalectomy as the standard of care for PA, future research should focus on cost-effectiveness analyses and comparisons with conventional laparoscopic techniques across diverse patient populations and surgical settings. This will help determine whether the benefits of robotic surgery justify its higher costs and resource demands, particularly in resource-constrained healthcare systems.

The adoption of robotic-assisted laparoscopic adrenalectomy, while offering technical advantages, raises important considerations regarding cost-effectiveness given the higher costs associated with robotic surgery compared to conventional laparoscopy or open approaches. These costs include the robotic system acquisition, maintenance, and disposable instruments, which may pose challenges in resource-constrained healthcare settings. However, the observed benefits in our cohort—such as high clinical and biochemical success rates, minimal complications, and shorter hospital stays—suggest potential cost savings through reduced postoperative care and improved patient outcomes, particularly in young patients with PA who face long-term morbidity from untreated disease. To date, specific cost-effectiveness analyses for robotic adrenalectomy in PA are lacking, unlike in other fields such as prostatectomy. Future studies should include cost-effectiveness analyses comparing robotic adrenalectomy to conventional laparoscopy and medical therapy, incorporating metrics such as quality-adjusted life years (QALYs) and long-term healthcare utilization, to determine whether the clinical benefits justify the increased upfront costs in diverse healthcare systems.

The cost-effectiveness of robotic adrenalectomy is a crucial consideration, as the procedure incurs a higher upfront institutional cost compared to laparoscopic adrenalectomy and open adrenalectomy due to capital investment and disposable instrument fees. However, the economic analysis for PA must consider the long-term perspective: the documented high success rate of adrenalectomy in achieving biochemical and clinical cure provides substantial lifetime cost savings by minimizing the need for expensive lifelong antihypertensive polypharmacy and preventing associated cardiovascular events. Furthermore, the technical advantages of RA—including enhanced visualization and dexterity—may mitigate the higher surgical costs by leading to superior perioperative outcomes such as zero conversions and shorter lengths of stay, thereby reducing the overall episode-of-care expenses and lowering morbidity risk. Given the variability in reported costs and the lack of high-level, definitive studies, we acknowledge that formal cost-effectiveness analyses incorporating quality-adjusted life years (QALYs) are still needed to precisely define the threshold volume and patient population for which the adoption of the robotic approach is economically superior to the established laparoscopic approach.

This study has several limitations that should be acknowledged. First, although our cohort of 18 patients provides valuable insights into the safety and efficacy of robotic-assisted laparoscopic adrenalectomy for small aldosterone-producing adenomas, the relatively small sample size represents a key limitation. This restricts the generalizability of our findings and limits the statistical power for subgroup analyses or robust identification of independent predictors of outcomes, such as factors influencing complete clinical success or operative metrics. Larger, multicenter cohorts, such as those in the Primary Aldosteronism Surgical Outcome (PASO) studies involving hundreds of patients, would enhance the reliability and broader applicability of results. Future collaborative efforts are warranted to address this and validate our observations in diverse populations. Second, the study was conducted at a single center by a single, highly experienced robotic surgeon, which may not reflect outcomes achievable in centers with less robotic expertise or lower surgical volumes. Moreover, the single-arm, prospective design without a control group (e.g., conventional laparoscopy or medical therapy) makes it difficult to attribute outcomes solely to the robotic technique. Results may be influenced by surgeon expertise or patient selection bias. Additionally, AVS, the gold standard for lateralization, was not performed in all patients due to technical limitations and availability; reliance on imaging for surgical decision-making in some cases may have introduced selection bias. Future multicenter studies with larger cohorts, longer follow-up, and comparative arms are needed to validate these findings and assess the reproducibility of robotic adrenalectomy outcomes in broader clinical settings.

## 5. Conclusions

Our study demonstrated excellent clinical outcomes with robotic-assisted laparoscopic adrenalectomy for small, aldosterone-producing adrenal adenomas. We advocate for screening patients with uncontrolled hypertension for primary hyperaldosteronism, as this condition is often treatable. In cases of clinically confirmed unilateral adenoma, we recommend considering robotic-assisted laparoscopic adrenalectomy as the treatment of choice.

## Figures and Tables

**Table 1:** Patient baseline demographic and clinical data.

Variable	Description (*n* = 17)
Gender, *n* (%) Male Female	13 (76.5.5) 5 (23.5)
Median Age, years (IQR)	53 (44–65)
Median BMI, kg/m^2^ (IQR)	27 (24–31.6)
Diabetes, *n* (%)	5 (29.4%)
Smoker, *n* (%)	2 (11.7%)
Median adenoma size, mm (IQR)	15 (10–28)
Median preoperative antihypertensive medication, *n* (IQR)	3 (2–4)
Pre-operative adrenal vein sampling, *n* (%)	3 (17.6)

BMI—Body Mass Index; IQR—Interquartile Range; mm—Millimeter.

**Table 2:** Postoperative outcomes.

Variable	Description (*n* = 17)
Length of postoperative hospital stay, median *n* (IQR)	3 (3–4)
Clinical success, *n* (%)	13 (76.5)
Partial success, *n* (%)	4 (23.5)
Failure, *n* (%)	0

## Data Availability

The data presented in this study are available on request from the corresponding author. The data are not publicly available due to privacy restrictions.

## Supplementary Materials

The following supporting information can be downloaded at https://www.mdpi.com/article/10.3390/jcm15010173/s1. Figure S1: Robotic port placement for left-sided adrenalectomy.
