# Supplementary figures and images for: Adrenalectomy as a Treatment Option for Primary Aldosteronism in the Era of Robotic-Assisted Surgeries—Is It Time to Use It More Often?
Source: J Clin Med. 2025 Dec 25;15(1):173. doi: 10.3390/jcm15010173 (PMC12787028; doi:10.3390/jcm15010173)

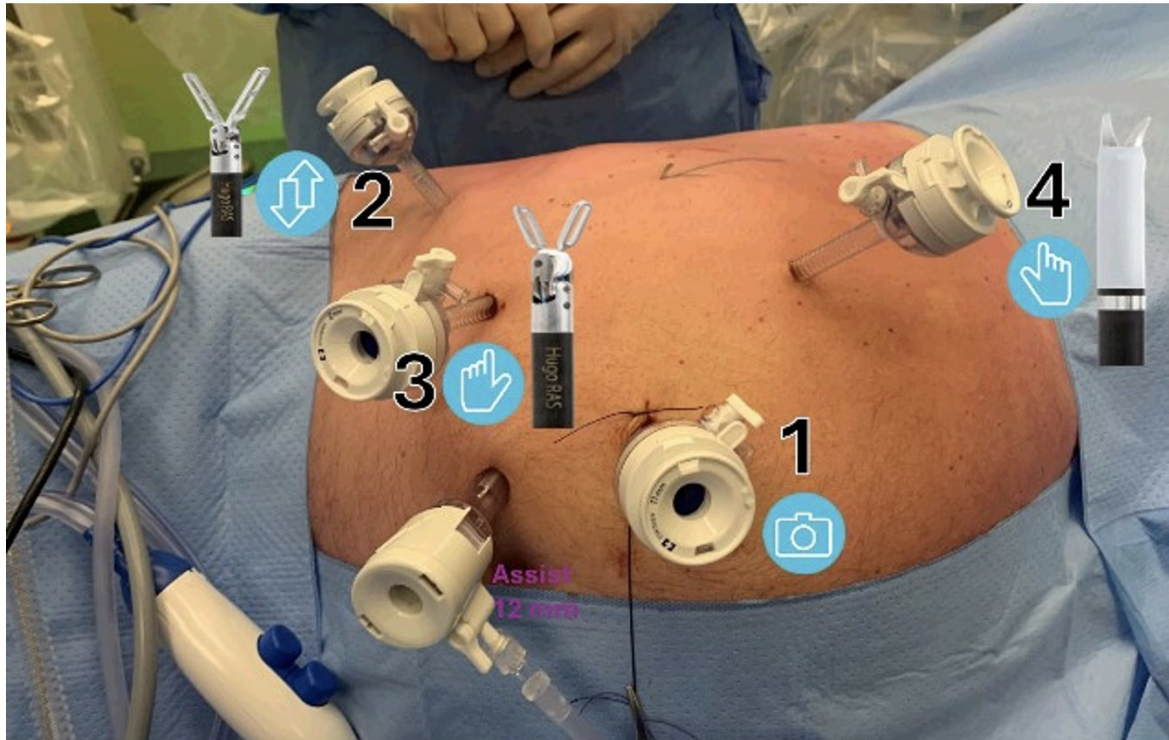

**Figure S1.** Robotic port placement for left-sided adrenalectomy.

Supplement: Supplementary file 1 [file jcm-15-00173-s001.zip › jcm-4037871-supplementary.pdf]
